# Enhanced Postbiotic Metabolite GABA Production in Skim Milk Using *Weissella cibaria* UF-274 and Whole-Genome Analysis

**DOI:** 10.3390/metabo16030175

**Published:** 2026-03-06

**Authors:** Ida Bagus Agung Yogeswara, Ni Wayan Nursini, I Gusti Ayu Wita Kusumawati, Rusli Fidriyanto, Dietmar Haltrich

**Affiliations:** 1 Department of Nutrition, Universitas Dhyana Pura, Jl Raya Padangluwih, Tegaljaya, Kuta Utara 80361, Bali, Indonesia; nursini@undhirabali.ac.id (N.W.N.); witakusumawati@undhirabali.ac.id (I.G.A.W.K.); 2 Center of Excellent for Nutribiome, Universitas Dhyana Pura, Jl Raya Padangluwih, Tegaljaya, Kuta Utara 80361, Bali, Indonesia; 3 Research Center for Applied Zoology, National Research and Innovation Agency, Jl. Raya Jakarta-Bogor Km 46, Cibinong, Bogor 16911, West Java, Indonesia; rusl006@brin.go.id; 4 Laboratory of Food Biotechnology, Department of Biotechnology and Food Science, Universität für Bodenkultur (BOKU)-University, 1190 Vienna, Austria; dietmar.haltrich@boku.ac.at

**Keywords:** GABA, *Weissella cibaria*, whole genome, lactic acid bacteria, fermentation

## Abstract

**Background/Objectives:** Gamma-aminobutyric acid (GABA) is a bioactive, non-proteinaceous amino acid with potential health benefits. *Weissella cibaria* UF-274 is an important lactic acid bacterium isolated from Balinese fermented sausage (urutan) with GABA-producing abilities. The aim of this study was to enhance GABA synthesis in skim milk as a basal substrate, as well as whole genome sequencing and analysis to evaluate the functionality and safety of the strain. **Methods**: A Box–Behnken response surface design was used to enhance GABA accumulation in skim milk. **Results:** The optimum conditions for GABA production were at concentrations of glucose of 23.91 g/L, monosodium glutamate concentrations of 2.32 g/L and pyridoxal-5′-phosphate at 46 μM. The genome assembly produced a high-quality draft with a 2.53 Mb circular chromosome and 2378 coding sequences. A whole genome analysis revealed that the strain possesses a glutamine amidotransferase (puuD-like) as an alternative route linked to the GABA pathway. AntiSMASH prediction results showed that the strain has two biosynthetic gene clusters including terpene and type III polyketide synthases. Several bioinformatic approaches predicted no antibiotic resistance genes, while van genes encoding vancomycin resistance were detected with low pathogen risk with one approach. **Conclusions**: *Weissella cibaria* UF-274 is a promising GABA producer with genomic evidence and a good candidate for functional food development.

## 1. Introduction

Gamma-aminobutyric acid (GABA) is recognized as a primary inhibitory neurotransmitter within the mammalian central nervous system. In plants and microorganisms, GABA functions as a defense mechanism, being excreted under stress conditions [1]. This non-proteinaceous amino acid exhibits well-documented physiological effects in humans, including the modulation of neuronal excitability and potential benefits for blood pressure regulation, obesity management, and stress reduction [2,3,4]. GABA biosynthesis primarily occurs through the decarboxylation of L-glutamate, a reaction catalyzed by L-glutamic acid decarboxylase (GAD; EC 4.1.1.15), an enzyme encoded by the *gad*A or *gad*B genes [5]. This enzymatic reaction necessitates a cofactor, pyridoxal-5′-phosphate, to initiate the decarboxylation. Following synthesis, the *gad*C-encoded glutamate/GABA antiporter is responsible for secreting GABA from the cell [6,7]. Nevertheless, certain microbial species, including *Escherichia coli* and *Aspergillus oryzae*, utilize an alternative glutamine-putrescine pathway involving a series of enzymes to convert arginine, ornithine, and agmatine into GABA [8,9]. The specific metabolic pathways employed for GABA synthesis vary across different organisms, with bacteria often utilizing distinct enzymatic cascades compared to fungi or plants.

Food-based delivery of GABA has attracted increasing interest, as fermentation by lactic acid bacteria (LAB) offers a pathway to naturally enriched functional foods without the need for chemical additives [10]. In contrast, chemical synthesis can lead to unwanted by-products. LAB are recognized as efficient cell factories for GABA production and generally hold the Generally Recognized As Safe (GRAS) status. Several GABA-producing LAB species and strains, including *Lactiplantibacillus plantarum*, *Lactobacillus brevis*, *Lactobacillus paracasei*, *Lactobacillus pentosus*, and *Lactococcus lactis*, have been identified [11,12,13,14,15]. Traditional fermented foods serve as a rich reservoir of unique LAB species and strains possessing adaptive traits. Several GABA-producing LAB have been isolated from various fermented foods like kimchi [16,17], fermented fish [18,19], fermented soybean [20,21], fermented sausages [22], and fermented milk [23]. Among these, LAB species/strains showed variability in GAD yield and characteristic in food matrix applications, suggesting a highly strain-dependent flexibility for food applications.

In this study, the strain *Weissella cibaria* UF-274 was isolated from Balinese fermented sausage (*urutan*) and shown to possess GABA-producing ability. A niche artisanal matrix often selects for strains with distinctive metabolic and stress-resistance characteristics relevant to food fermentation. However, the utilization of these strains for other food products necessitates applied optimization within a target matrix. Skim milk, for instance, offers a commercially relevant, and cheap substrate where protein and mineral composition can significantly influence microbial metabolism [24,25,26]. While *W. cibaria* has indeed emerged as a novel GABA-producing LAB, its promising potential as well as its full ability for GABA production has not yet been thoroughly investigated, particularly within different food systems. This gap in knowledge suggests a novel avenue for further investigation. The synthesis of this metabolite is primarily affected by fermentation parameters such as the supplementation of carbon sources, nitrogen sources and cofactors, as well as by the incubation temperature and incubation time. Furthermore, the specific supplementation of various nutrients in the medium of a food matrix can significantly influence GABA yield. Consequently, it is important to investigate the optimum nutrient conditions for GABA production in *W. cibaria* UF-274.

Whole genome sequencing (WGS) is crucial for empirically optimizing GABA production in *W. cibaria* UF-274. It enables the detection of GABA synthesis pathways, comprehensive genomic characterization, and the assessment of functional characteristics like secondary metabolite production [27,28]. Furthermore, WGS facilitates the prediction of antimicrobial resistance or virulence determinants, which are essential for compliance with food safety regulations [29,30]. In this preliminary study, we evaluate the potential of this strain as a starter or adjunct starter in skim milk as a basal substrate in addition to WGS-based analysis to maximize GABA yield under food-relevant conditions and establish genomic evidence for biosynthetic potential and strain safety.

## 2. Materials and Methods

### 2.1. Taxonomy Profiles in Fermented Urutan

Fermented *urutan* was obtained from a local household located in Baturity, Tabanan regency, Bali, Indonesia. The fermented sausages were vacuum packed and stored at 4 °C prior to metagenomic analysis. Analysis of the bacterial composition in fermented *urutan* was conducted using the genomic extraction kit DNA Miniprep (Zymo Research, D4300, Irvine, CA, USA) according to the manufacturer instructions and subsequent sequencing. The concentration of the extracted DNA was measured using a NanoDrop spectrophotometer (Thermo Fisher Scientific, Waltham, MA, USA), and it was stored at –20 °C until further analysis. For identification at the species level, long-read sequencing targeting 16S rRNA was performed. The gene was amplified using the forward primer 27F: 5′—AGAGTTTGATCMTGGCTCAG—3′ and the reverse primer 1492R: 5′—GGTTACCTTGTTACGACTT—3′. Subsequently, barcoding the PCR process was done using the PCR barcoding expansion kit 1-96 (Nanopore, Oxford, UK), followed by purification of the PCR products. An adapter-binding PCR was performed by ligation using a Ligation Sequencing Kit (Nanopore, Oxford, UK). The purified product was loaded into the Flow Cell MinIon (Nanopore) device operated by MinKNOW software version 22.05.7 (Nanopore) [31].

### 2.2. Isolation, Screening and Identification of GABA-Producing LAB

Ten grams of fermented *urutan* were suspended in 90 mL of sterile 0.85% NaCl solution and homogenized. This was followed by serial 10-fold dilutions and spreading on de Man Rogosa and Sharpe (MRS) agar supplemented with 0.5% (*w*/*v*) monosodium glutamate (MSG, Ajinomoto, Indonesia) and bromocresol purple (BCP). The plates were incubated at 37 °C for 24–48 h. LAB isolates with GABA-forming potential were selected based on the appearance of a yellow-clear zone around the colonies. The selected isolates were streaked on the same medium to obtain pure colonies. The purified colonies were stored at −80 °C in a 30% glycerol solution for further analysis.

To determine GABA production, each LAB isolate was inoculated in MRS broth medium containing 0.5% MSG and incubated at 37 °C for 24 h. The cultured MRS medium was then centrifuged at 8000× *g* for 5 min at 4 °C, and 1–2 μL of the supernatants was spotted onto silica plates 60 F_254_ (Merck, Darmstadt, Germany). The plates were placed in a chamber filled with the mobile phase containing a mixture of 1-butanol:acetic acid:aquadest (5:2:2). After separation, spots were visualized with 0.5% (*w*/*v*) ninhydrin and heated at 100 °C for 5 min. LAB with GABA-producing ability were further identified, and the amount of GABA was quantified using HPLC [32].

DNA barcoding of 16S rRNA was performed to identify GABA-producing LAB using the GeneJET DNA kit (Thermo Fisher, MA, USA). The isolated genomic was amplified using universal primers 27F: 5′—AGAGTTTGATCMTGGCTCAG—3′ and reverse primer 1492R: 5′—GGTTACCTTGTTACGACTT—3′. The partial sequences of 16S rRNA of each LAB were compared with the GenBank (NCBI) database, followed by the construction of a phylogenetic tree using MEGA software (v. 4).

### 2.3. Effect of Nutrients on GABA Production

Skim milk was sterilized and inoculated with 0.5% (*v*/*v*) of an active culture of *W. cibaria* UF-274. The skim milk was fermented at 37 °C for 24 h. Based on preliminary studies, glucose and monosodium glutamate (MSG) were selected as carbon and nitrogen sources. The effects of glucose concentration, MSG concentration and pyridoxal-5′-phosphate (PLP) concentration on GABA production in skim milk were determined. The experimental range of the three factors studied was as follows: glucose concentrations were set up at 0–40 g/L, MSG concentrations at 0–3 g/L, and PLP concentrations were at 0–60 μM. All experiments were performed in triplicate.

To obtain optimum conditions for GABA production, a Box–Behnken test design with three factors was set up using Stat Ease 360 v25. 0.1 (Stat Ease Inc., Minneapolis, MN, USA). The results were analyzed using ANOVA and response surface plots. Optimal conditions for GABA formation were determined by analyzing response surface plots from the response surface model (RSM).

### 2.4. GABA Quantification

GABA concentration in the supernatant was quantified using High Pressure Liquid Chromatography (HPLC, Thermo Dionex Ultimate 3000, Thermo Fisher, MA, USA). Fifty μL of supernatant was derivatized using 300 μL of an o-phthalaldehyde (OPA) solution (containing 1 mL methanol, 4 mL borate buffer pH 9.0 and 30 μL 2-mercaptoethanol). The derivatized samples (20 μL) were injected into the HPLC with a mobile phase consisting of gradient A (CH_3_OH; 50 mM natrium acetate: tetrahydrofuran pH 6.8) and gradient B (65% CH_3_OH). The system was operated at a flow rate of 1.5 mL/min with an excitation wavelength at 300 nm and emission wavelength at 500 nm. GABA standard solutions were prepared in various concentration (0–60 mg/L) and fitted by a standard curve (y = 9280x – 85,753, R= 0.9946) [33].

### 2.5. Whole Genome Analysis of W. cibaria UF-274

The whole genome sequencing of strain UF-274 was carried out using Oxford Nanopore Technology (ONT). The gDNA samples were used as the input for library preparation using the Library Preparation kit from Oxford Nanopore Technologies. gDNA was repaired using an end prep enzyme mix, generating DNA with 5′-phosphorylated, 3′-dA-tailed ends. The repaired DNA was ligated with an ONT-compatible adapter. The library was quantified with a Qubit Fluorometer before loading onto the flow cell. Sequencing was performed using PromethION (ONT) until the desired yield was achieved. The quality of sequencing was filtered with Nanoplot. Read correction and assembly were performed using Canu (v2.2) and Flye (v2.8), respectively. The assembled sequence was polished four times with Racon and three times with Medaka. Mapping was performed using minimap2. The quality of the assembled sequence was determined using Quast (v5.3.0) and Qualimap (v2.3). Annotation and visualization were conducted using Rast, Prokka (v1.14.5) and Genovi (v0.4.3), respectively. Additional Gene Ontology (GO) and Kyoto Encyclopedia of Genes and Genomes (KEGG) annotation were performed using EggNOG-mapper, while Biosynthetic Gene Cluster (BGC) analysis was done using AntiSmash (v8.0) [28,34,35].

Safety assessment of *W. cibaria* UF-274 was performed using the ResFinder v4.60 software and Resistance Gene Identifier (RGI) tool v6.0.3 software in the Comprehensive Antibiotic Resistance Database (CARD). Genes linked to resistance to different classes of antibiotics were analyzed. The RGI results were classified into three types: “Perfect hit” (100% identical with reference), “String hit” (bit score > 450 and not identical), and “Loose hit” (bit score < 450, matched in some regions). In addition, ResFinder can also be used to predict antimicrobial resistance genes (AMR) with a 90% threshold and a 60% minimum length. The pathogenicity of the strain was assessed using PathogenFinder (v0.6.0), with the addition of prophage region, Clustered Regularly Interspaced Palindromic Repeats (CRISPRs) and insertion sequences using Proksee server and PHASTER.

## 3. Results 

### 3.1. Bacterial Composition in Fermented Urutan

**Figure 1:**
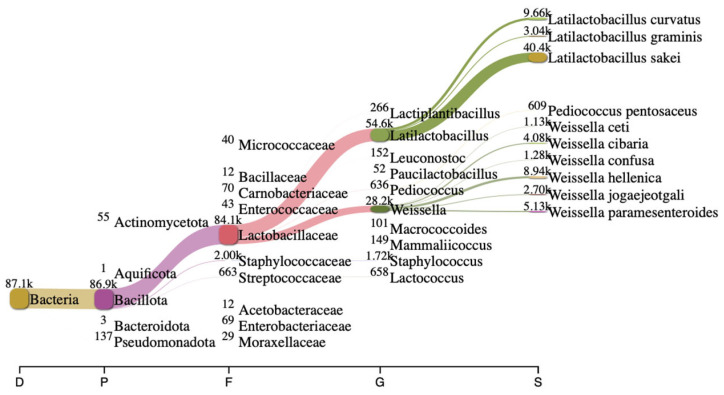
Bacterial composition in fermented *urutan*. Sankey plot analysis showing different communities at different hierarchical taxonomies in fermented *urutan*.

Long-read 16S rRNA sequencing using Next-Generation Sequencing (NGS) is a powerful tool to analyze bacterial composition within communities with a broader profile for species-level identification (Figure 1). In this study, the bacterial composition of artisanal *urutan* revealed a community structure dominated by Firmicutes, with lactic acid bacteria (LAB)—primarily *Weissella, Lactococcus, Lactobacillaceae, Latilactobacillus* and *Leuconostoc—*representing the dominant functional group. At the species level, *W. hellenica* (8940) was dominant, followed by *W. paramesenteroides* (5130) and *W. cibaria* (4080), respectively. 

### 3.2. Screening of GABA-Producing Lactic Acid Bacteria

**Figure 2:**
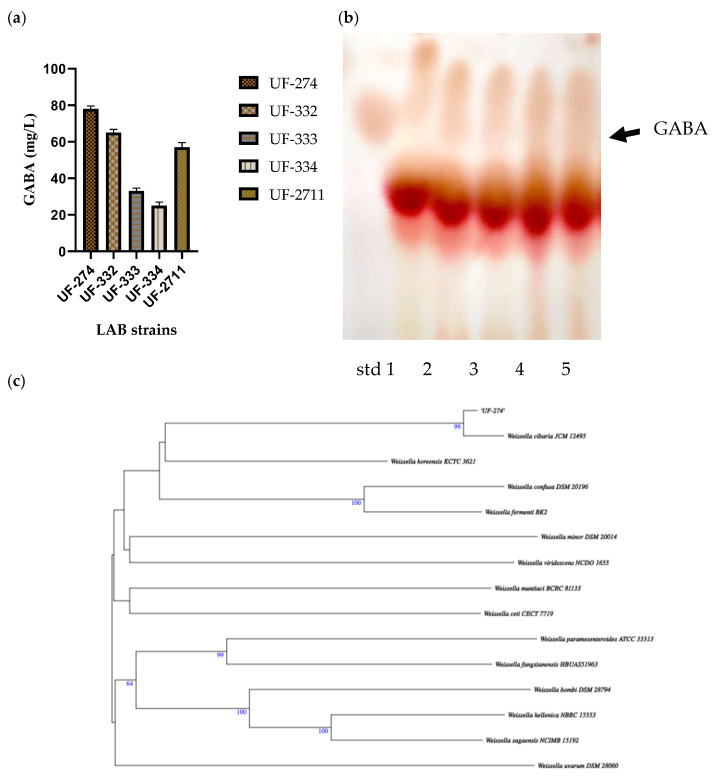
GABA production in strains *W. cibaria* (UF—274, 332, 334, 2711) and *L. sakei* UF-333 (**a**), TLC screening of their GABA-producing ability, lane 1–5; UF-274, 332, 333, 334, 2711 (**b**), and (**c**) analysis of the phylogenetic tree of *W. cibaria* UF-274 using barcoding 16S rDNA, the tree was constructed using MEGA X software (v. 4).

Thirty-two isolates were obtained and screened for their ability to perform GABA synthesis. Five strains were identified as positive GABA producers when using TLC (Figure 2). Quantitative analysis showed that the ability of the strains to produce GABA varied depending on the strains and species, and the best producing strain was UF-274. Strain UF-274 produced 78 mg/L of GABA during 24 h of cultivation in MRS broth medium supplemented with 1% MSG. Other good producers are strains UF-332 and UF-2711 with GABA production of 65.08 mg/L and 56.76 mg/L, respectively. All strains were identified using 16S rDNA barcoding. Four strains (UF-274, UF-332, UF-334, UF-2711) were *Weissella cibaria* species and one strain, UF-333, was identified as *Lactobacillus sakei.* Based on a phylogenetic tree, strain *W. cibaria* UF-274 showed 99% similarity to strain *W. cibaria* JCM 12495. In addition, this result aligned with a Sankey plot analysis (Figure 1), indicating that the diagram provides an integrated visual confirmation that *Weissella* is a dominant, culturable taxon in fermented *urutan*, and culturing successfully retrieved a functionally relevant strain (UF-274) responsible for the observed GABA phenotype. 

### 3.3. Optimization of GABA Production in Skim Milk

To optimize GABA production in skim milk, the three factors glucose, monosodium glutamate and PLP addition to skim milk were selected as independent variables and studied for their effect on GABA enhancement. A Box–Behnken design was used to analyze these variables and to determine the optimum concentrations. A total of sixteen experiments with different concentrations of glucose, MSG and PLP was conducted in triplicate.

The proposed model obtained from the experimental results is shown in Table 1, with the adjustment coefficient value of 0.9251, indicating that the high correlation coefficient (R^2^) confirms the model reliability for identifying and forecasting the optimal medium composition. The F-value was 8.23, which again suggests that the model is statistically significant, and the *p*-value was 0.0092. *p*-values of less than 0.0500 indicate that model terms are significant. In this case B, A^2^, B^2^ are significant model terms. The equation in terms of coded factors can be used to make predictions about the response for the given levels of each factor. By default, the high levels of the factors are coded as +1 and the low levels are coded as −1. The coded Equation (1) is useful for identifying the relative impact of the factors by comparing the factor coefficients. The final equation in terms of coded factors is presented in Equation (1):

GABA = +6.28 − 0.1865A + 1.94B + 0.1554C − 0.4690AB − 0.7174AC + 0.6199BC − 1.73A^2^ − 1.11B^2^ − 0.8055C^2^. (1)

**Figure 3:**
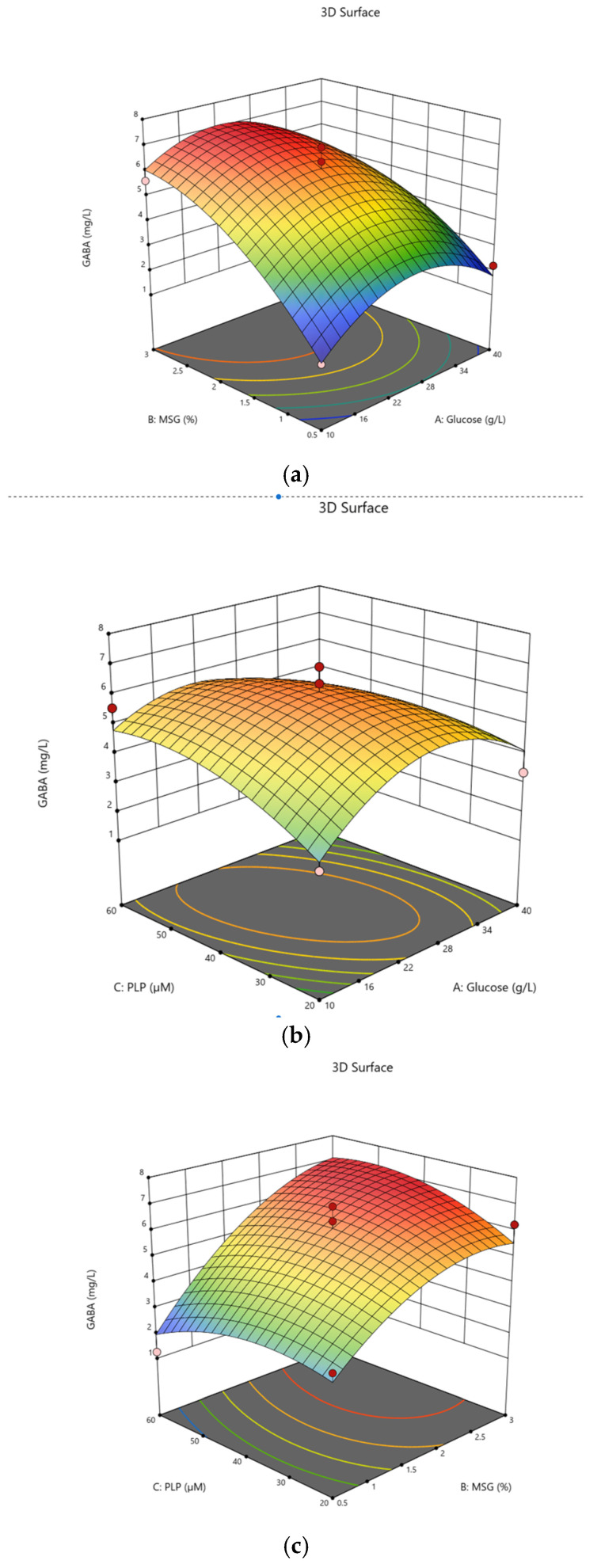
Contour lines and the response surface for the GABA production in skim milk; (**a**) GABA production with combination of glucose and MSG; (**b**) PLP and glucose and (**c**) combination of MSG and PLP.

The equation can be used to predict GABA production, where A, B and C code for glucose, MSG and PLP concentrations, respectively. Response surface plots were used to display the interaction amongst glucose, MSG and PLP in skim milk. The addition of glucose slightly increases GABA content in skim milk (Figure 3a). The effects of PLP and glucose on GABA production are presented in Figure 3b. Higher levels of PLP did not significantly affect the GABA content. The effects of MSG and PLP showed a significant interaction (Figure 3c). The availability of PLP suggests that the cofactor modulates GAD activity to convert MSG to GABA.

The optimum conditions for GABA synthesis according to RSM are a glucose concentration of 23.91 g/L, MSG at 2.32 g/L and PLP at 46 μM. The predicted GABA production under these optimal conditions is 7.040 mg/L. Verification of the actual GABA production was determined under optimal conditions with GABA content of 7.023 mg/L, suggesting that the predicted value is in excellent agreement with the actual experimental value. 

### 3.4. Genomic Characteristic of W. cibaria UF-274

**Figure 4:**
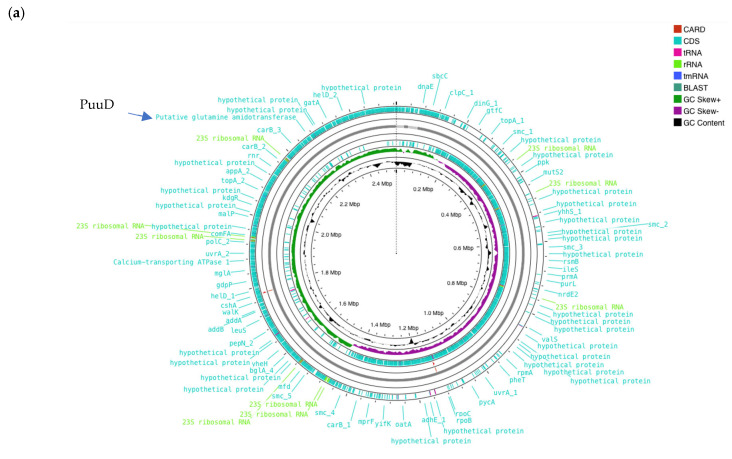

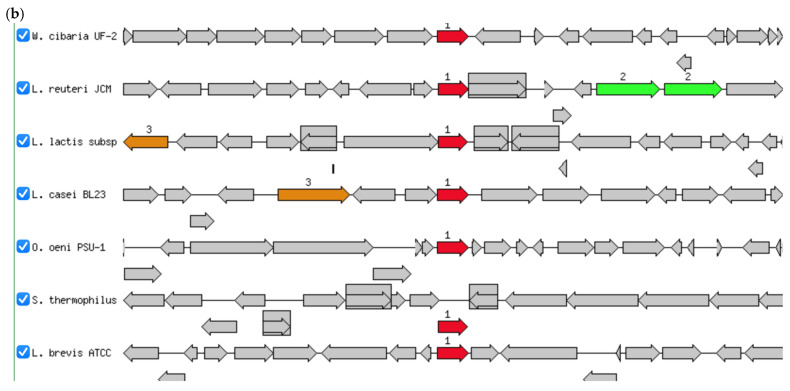
(**a**) Circular genome of *W. cibaria* UF-274. From outer to inner rings, open reading frame (ORF), GC content, GC skew – GC skew +. The red lines represent tRNA, green lines represent rRNA and blue lines represent tmRNA. (**b**) Comparative gene neighborhoods surrounding the glutamine amidotransferase (GATase; red arrows) in *Weissella cibaria* UF—274 and representative lactic acid bacteria. Genomes were annotated using RAST system. Sets of genes with similar sequence are grouped with the same number and color. Genes whose relative position is conserved in at least four other species are functionally coupled and share gray background boxes. Green arrows indicating alpha glucosyltransferase; brown arrows indicating pyruvate oxidase.

The whole genome sequencing of *W. cibaria* UF-274 generated a high-quality draft genome with a total length of 2.53 Mb and a GC content of 44.86% (Figure 4a). The genome consists of four contigs with the assembly quality as reflected by a contig N50 of 2.46 Mb and an L50 value of 1. Genome annotation identified 2378 coding sequences (CDS), of which 1577 genes (66.3%) were assigned putative functions, while 864 sequences were classified as hypothetical proteins (Table 2). The genome also encoded 87 tRNA genes and 28 rRNA genes, indicating a well-represented translational machinery typical for lactic acid bacteria. Benchmarking Universal Single-Copy Orthologs (BUSCO) v.5.8 and CheckM was performed to assess the assembly completeness with values of 96% and 98.3% completeness, respectively, with a contamination level of 0.72%.

Of particular interest, annotation using RAST, Prokka and EggNOG revealed enzymes potentially involved in GABA metabolism. Genomic region centered on PuuD-like glutamine amidotransferase (GATase) family identified in UF–274 assembly (Figure 4b). The comparative neighborhoods map shows that homologous GATase–encoding genes are present in several LAB species. Although the neighboring genes differ between species, the GATase is consistently located in the same chromosomal region, suggesting it is part of a conserved metabolic module rather than a strain–specific insertion.

### 3.5. Functional Properties of Annotated Genes

**Figure 5:**
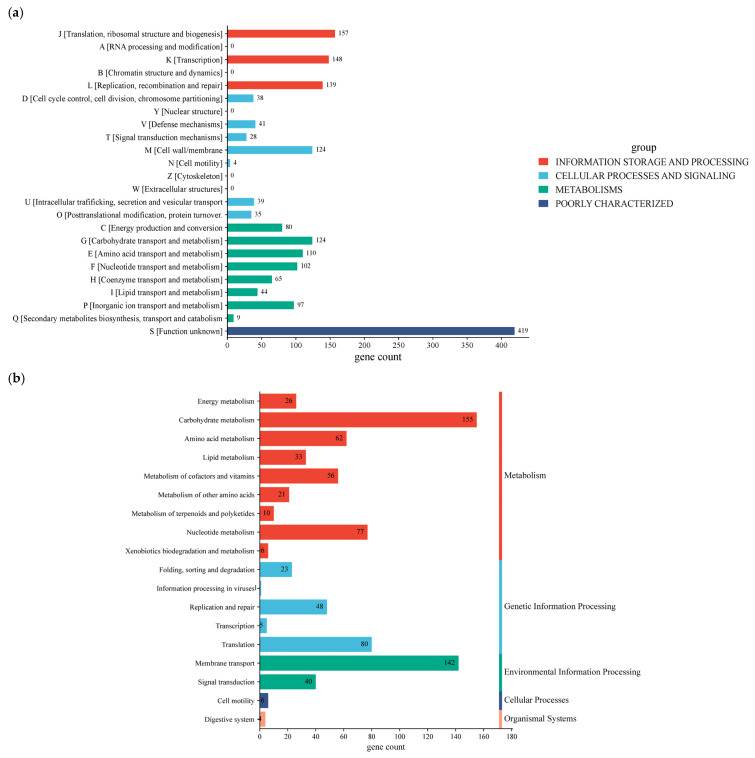

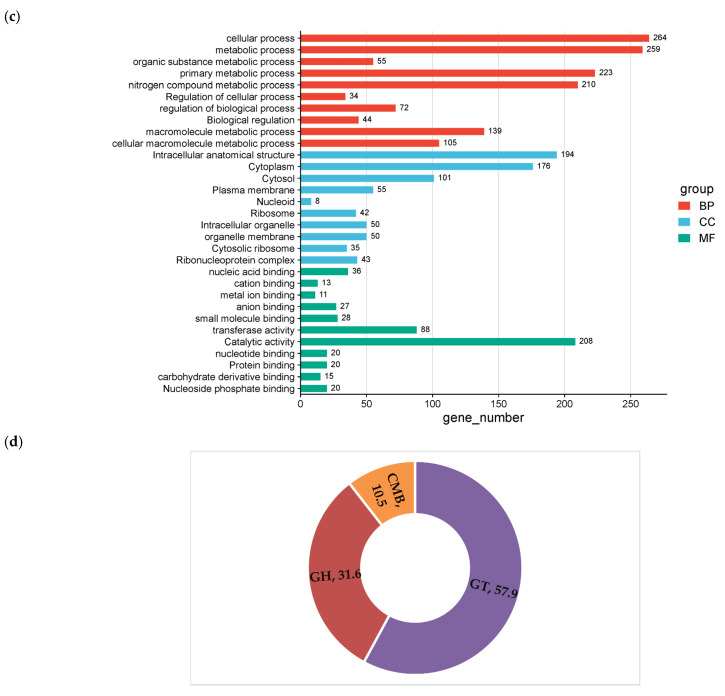
COG functional annotations (**a**); KEGG functional annotations (**b**); GO annotation of *W. cibaria* UF-274 (**c**); Carbohydrate-active enzymes annotation (**d**).

The functional properties of annotated genes were compared against the Clusters of Orthologous Group (COG) and the Kyoto Encyclopedia of Genes and Genomes (KEGG) databases (Figure 5a). Based on annotated gene classification, the chromosome of *W. cibaria* UF-274 classified into 18 categories, in which 446 genes in total are associated with metabolism, of which 155 genes are linked to carbohydrate metabolism, followed by amino acid metabolism (62 genes), nucleotide metabolism (77 genes), and cofactor as well as vitamin metabolism (56 genes), respectively. In addition, strain *W. cibaria* UF-274 revealed 156 genes and 148 genes responsible for genetic information processing and environmental information, respectively. Eighty genes are associated with the translation process and 142 genes are related to membrane transport of *W. cibaria* UF-274. Functional groups based on COG consist of “function unknown” [S] with 419 genes (17.6%), followed by genes that mostly associate with core information processing functions with 444 genes and cellular signaling with 309 genes. Genes assigned to Q (secondary metabolite biosynthesis) were few (nine genes), consistent with in silico antiSMASH detection of two relatively small BGCs (terpene and type-III PKS) in strain UF-274. 

KEGG annotation results showed that the strain UF-274 has an active carbohydrate metabolism (155) with the highest results, followed by amino acid (62) and nucleotide metabolism (77), respectively (Figure 5b). In the GO analysis of the strain (Figure 5c), the majority of biological processes are in the category of cellular (264 genes) and metabolic processes (259 genes). The molecular function of the strain is dominated by catalytic activities (208 genes) and transferase activities (88 genes). In addition, the majority of the cellular components consist of intracellular anatomical structures (194) and the cytoplasm (176). A total of 52 proteins were identified as carbohydrate-active enzymes (CAZymes), resulting in a total of 57 CAZy family annotations (Figure 5d). Glycosyltransferases (GT) represent the most abundant class (57.9%), followed by glycoside hydrolases (GH) (31.6%). A clear predominance of GT over GH is commonly associated with cell wall biosynthesis, extracellular polysaccharide production and glycan restructuring rather than degradation of complex carbohydrates. A smaller proportion of carbohydrate-binding modules (CBM) was also detected (10.5%). The dominance of GT families, particularly GT1, GT4, and GT2, suggests that this organism possesses a strong capacity for glycan biosynthesis and carbohydrate modification rather than extensive polysaccharide degradation.

### 3.6. Biosynthetic Gene Clusters and Safety Analysis

**Figure 6:**
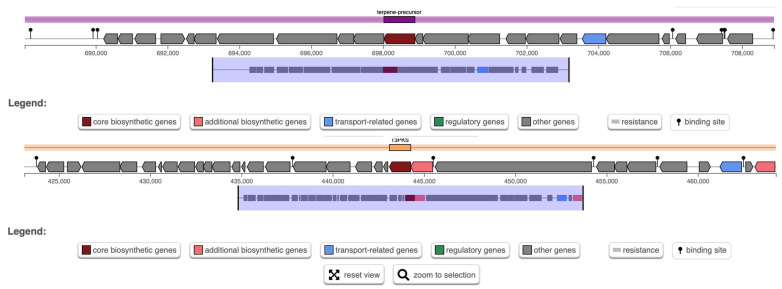
Analysis of biosynthetic gene clusters in *W. cibaria* UF-274 genome. Prediction of BCG using AntiSMASH.

AntiSMASH analysis predicted two biosynthetic gene clusters (BGCs) in *W. cibaria* UF-274: a terpene cluster and a type-III polyketide synthase (T3PKS) cluster (Figure 6). The T3PKS region (region 4.1) encodes, in addition to the core polyketide synthase (PKS) enzyme, proteins with predicted hydrolase and ligase activities, including a methionine aminopeptidase (MetAP) and a hydroxymethylglutaryl-CoA (HMG-CoA) synthase. The terpene biosynthetic gene cluster (BGC) (region 4.2) contains core genes consistent with phytoene/carotenoid biosynthesis and predicts the potential to synthesize multiple classes of terpenoids, including sesquiterpene, diterpene, sesterterpene and triterpene precursors. Bacterial terpenes are assembled from universal prenyl diphosphate substrates (GPP, FPP, GGPP) by terpene synthases that catalyze ionization and rearrangement to generate diverse skeletons

To assess the safety of the strain, the presence of antibiotic resistance genes was analyzed using ResFinder and CARD. ResFinder analysis did not detect any antibiotic resistance genes in the *W. cibaria* UF-274 genome, suggesting the safety of the strain as it cannot transfer antibiotic resistance gene. In contrast, CARD analysis revealed that the strain harbors *van*T and *van*Y genes, encoding for vancomycin resistance. These genes are located on the complement strand in the genome with a length ranging from 1,064,635 to 1,065,420 bp. The product of the genes is D-alanyl-D-alanine carboxypeptidase, commonly found in the bacterial membrane or secreted into periplasmic space. 

The genomic stability of the strain was assessed by the presence of prophages in the genome of UF-274. PHASTER analysis showed two prophage regions in the genome with intact prophage regions. These prophage regions have a GC content of 42.39% (region 1) and 42.31% (region 2), respectively. The prophage of each region is located in contig 4 and consists of 48 proteins in region 1 (region length 44.6 kb) and 44 proteins in region 2 (region length 37 kb). 

## 4. Discussion

This study demonstrates the potential of *Weissella cibaria* UF-274 as a GABA-producing strain adapted to an artisanal fermented-meat niche and evaluates its performance in a skim-milk matrix together with whole-genome characterization. In this study, *L. sakei* was found abundance in Balinese fermented *urutan*, as this species is considered a major bacterium in fermented sausages [36,37,38]. Minor populations of non-LAB Firmicutes (including *Staphylococcus* spp.) were consistently detected across various samples. The presence of *Staphylococcus* and *Lactobacillus* is considered as dominant microflora in several types of traditional fermented sausages, where *Staphylococcus* spp. becomes one of the sub-major species in most fermented sausages [39,40]. Such dominance is consistent with a high protein content, mild salt concentrations, and an anaerobic microenvironment of fermented sausages, which favors heterofermentative and facultative heterofermentative LAB adapted to amino acid and peptide metabolism [39]. A number of GABA-producing LAB were successfully isolated from Nham (Thai fermented meat) such as *Lactobacillus namurensis* HN8 and *Pediococcus pentosaceus* NH2, which are considered as good GABA-producers and starters for fermented Nham [41]. To the best of our knowledge, this is the first report of GABA-producing species of *W. cibaria* and *L. sakei* isolated from fermented sausages.

The interaction between MSG and glucose indicates a synergistic effect, where MSG provides the substrate for L-glutamic acid decarboxylase (GAD) [11,42]. Furthermore, glucose controls cellular energy and central carbon fluxes that affect intracellular glutamate pools and cofactor regeneration. Gradual increase of PLP apparently did not affect GABA synthesis. Although PLP serves as cofactor for GABA synthesis, higher PLP concentrations did not enhance enzyme activity or exhibit negative physiological effects on cell stability [32]. This was also previously shown in strain *L. plantarum* FNCC 260 and *L. plantarum* FRT7, where excessive PLP did not enhance GABA production in MRS broth media [32,43]. The interaction between PLP and MSG suggests a dependence of PLP uptake on regeneration of the cellular metabolic status driven by glucose. In this study, we also observed a substantial reduction in GABA production from 78 mg/L in MRS broth to 7.023 mg/L in skim milk. This can be attributed to several limiting factors such as the presence of free glutamate or glutamate bound in peptides. MRS contains readily available free glutamate, whereas skim milk primarily contains glutamate bound within casein proteins, resulting in limited substrate availability [12,44]. Furthermore, the strain’s proteolytic activity may not be sufficient to release adequate amounts of free glutamate. The strong buffering capacity of milk may also attenuate acid stress induction, which is known to regulate the L-glutamic acid decarboxylase system. In addition, the absence of PLP supplementation and the complex nutrient competition in milk likely further constrained GABA biosynthesis. These factors collectively explain the markedly lower GABA yield observed in the dairy matrix. A similar study by Kanokwan et al. (2023) showed that the strain *Lactobacillus futsaii* CS3 did not produce GABA in skim milk medium without MSG, whereas the same strain yielded 11 g/L when 2% MSG was added [45]. Likewise, Kim et al. (2022) found that *Lactobacillus plantarum* Y7 produced 15 μg/mL of GABA in MRS medium supplemented with MSG, and only 6.85 μg/mL of GABA in skim milk medium [46]. These results suggest that MSG supplementation is indispensable and severely limits GABA yields.

Functional annotation of the genome indicates that the strain exhibits robust growth and metabolic versatility under fermentative conditions. A wide array of carbohydrate metabolism genes (G) and cell envelope biogenesis genes (M) may confer diverse carbohydrate substrates and adaptation to technological performance in food matrices such as adherence or tolerance to osmotic/acid stress [27,47]. The relatively high number of amino acid transport and metabolism genes (E) could contribute to GABA biosynthesis, either through the GAD pathway or via alternative gamma-glutamyl/putrescine-linked pathways (PuuD-like amidotransferase) [48,49]. Notably, in the CAZymes database, the strain showed various enzymes involved in the synthesis and degradation of various carbohydrate. The presence of GH families indicates the capability to hydrolyze α-glucans and other oligosaccharides, suggesting moderate carbohydrate utilization potential. Additionally, the detection of CBM families supports the functional role of CAZymes by facilitating substrate recognition and binding, thereby enhancing enzymatic efficiency [50,51].

The genomic analyses reported an absence of canonical *gad*A/*gad*B homologues alongside the identification of a puuD-like glutamine amidotransferase and related gamma-glutamyl metabolism genes. Although classical *gad*A/*gad*B L-glutamic acid decarboxylase homologs were not detected, the genome harbored genes encoding glutamine amidotransferase class I (peptidase family 26) as well as a gamma-glutamyl-GABA hydrolase-like enzyme (puuD-like) [48]. The enzymes belonging to this latter family are known to cleave gamma-glutamylated intermediates, suggesting that strain *W. cibaria* UF-274 may utilize an alternative, gamma-glutamyl-based pathway for GABA biosynthesis. The gene orientation revealed that *W. cibaria* UF-274 harbors the glutamine amidotransferase gene (red arrow) within a conserved syntenic locus shared among lactic acid bacteria [48,49]. The glutamine amidotransferase (GATase) gene is broadly conserved in the core genomes of diverse LAB (Figure 4b). Each species carries a highly similar GATase at a syntenic locus with different neighbors. For example, *L. casei*, *L. brevis* and *L. reuteri* show adjacent polyamine/arginine transport genes, suggesting the region is near arginine/ornithine metabolism genes in those species. We also observed that the gene order varied between lineages and linkage to the central one-carbon/amino acid metabolism in several genomes (for example, association with other aminotransferases). These findings suggest that GATase is present in a conserved chromosomal locus across these LAB species but may vary in flanking genes implying an ancestral gene with lineage-specific rearrangements.

The consistent gene orientation and neighboring decarboxylase-like and transporter genes suggest that this amidotransferase functions as part of an amino acid catabolic operon, potentially catalyzing gamma-glutamyl bond cleavage in gamma glutamyl-GABA intermediate [48]. The absence of *gad*A/*gad*B homologs and the presence of this puuD-like amidotransferase (blue arrow in Figure 4a) indicate that *W. cibaria* UF-274 may utilize an alternative GABA-forming pathway distinct from the conventional GAD system. Furthermore, the functionality of this enzyme is similar to that of GAD but with distinct evolutionary origins or structural characteristics [49]. This arrangement represents a potentially novel genomic organization for GABA biosynthesis within the *Weissella* genus and warrants further biochemical validation.

AntiSMASH analysis showed that methionine aminopeptidases in region 4.1 are ubiquitous metallopeptidases, required for protein maturation and are often considered therapeutic targets for several diseases such as cancer, obesity and microbial infections [52,53]. Their presence in the cluster may reflect accessory or tailoring functions. HMG-CoA synthase provides a key precursor for the mevalonate/isoprenoid pathway and is associated with biosynthesis of isoprenoid-derived cellular components such as menaquinones, ubiquinones and carotenoids, as well as cell-wall-associated isoprenoids. The region also contains genes showing similarity to bryostatin-like polyketide loci (GenBank DQ889942.1), indicating possible production of complex polyketide scaffolds, although the product identity must be confirmed experimentally [54,55,56]. The combined presence of a T3PKS and terpene biosynthetic machinery suggests that *W. cibaria* UF-274 harbors biosynthetic potential for structurally diverse secondary metabolites that could influence aroma, redox balance, membrane chemistry or inter-microbial interactions in fermented foods [54].

Safety analysis of the strain showed that the resistance determinants located on the chromosome of LAB are usually intrinsic and lack linkage to mobile genetic elements, meaning they are unlikely to be transmitted horizontally to other bacterial species [57,58]. Vancomycin resistance is commonly found in various *Lactobacillus* spp. and is considered to be intrinsic [29]. A number of studies have revealed that *Weissella* spp. are intrinsically resistant to vancomycin [59,60,61,62]. A recent study by Lee et al. (2026) showed that two *W. cibaria* strains (LB13201, LB3206) possess homologs of *van*T and *van*Y with only 33% identity, which is below the threshold values. Furthermore, the two strains had minimum inhibitory concentration (MIC) ≥ 256 μg/mL suggesting that vancomycin resistance in these strains is intrinsic [63]. The presence of *van*T and *van*Y genes in the strain genome should be interpreted with caution and therefore further transfer risk analysis is required. For this reason, the presence of chromosomal resistance genes is generally viewed as posing a lower biosafety risk than resistance carried on plasmids or transposons. In addition, the risk of the strain being a pathogen was determined with a risk score of 0.043, which is below the threshold and thus the risk of being a human pathogen is considered low. Moreover, VirulenceFinder (v3.2.0) analysis did not detect any virulence factors in the genome. These results suggest that the strain possesses safety characteristics that agree with a function as a starter or adjunct starter for functional foods application.

The presence of prophage may affect the genome stability in bacteria. Most of the prophages were dominated by hypothetical proteins and phage-related proteins from the genera *Lactococcus* and *Enterococcus.* Intact prophages exhibit various structural and functional properties and are commonly found in bacteria that inhabit diverse ecosystems [11,64]. Intact prophages found in the bacterial genome also serve as resistance mechanisms under harsh conditions and phage infections. CRISPR analysis showed that only one candidate of a CRISPR region (confirmed) could be found in the genome. This CRISPR region is located on the left flanking sequence with a length of 2,460,442 bp. Unfortunately, the system did not manage to detect the Cas locus in the genome. This could be due to a complete lack of Cas genes, or because the Cas genes are too few/divergent to meet the detection threshold. Genomic surveys of 160 *Weissella* spp. have shown no Cas gene cluster in their genome [65,66], while others possess a type II-A system (Cas1, Cas2, Cas9, Csn2). This finding indicates that the absence of Cas genes in the genome is a recurring pattern in *Weissella* and related LAB. The CRISPR-Cas system serves as an immune system in bacteria and archaea by degrading foreign DNA that enters the cell. The presence of a CRISPR-Cas system gives the strain some beneficial properties such as tolerance to phage infections and contributes to genomic stability under harsh conditions [47]. 

## 5. Conclusions

This study revealed that strain *W. cibaria* UF-274 is a promising GABA-producing LAB. Optimization using Box–Behnken in skim milk media can be formulated to enhance postbiotic GABA production. A whole genome analysis revealed that the strain employs an alternative gamma-glutamyl-linked route rather than the *gad*A/*gad*B system for GABA synthesis. Further genome mining analysis also revealed two small BGCs, terpene and T3PKS, which may affect ecological traits in fermentations. Safety analysis found no evidence of mobile antibiotic resistance genes; however, van genes and two intact prophages were detected with low pathogen risk scores. Thus, the safety of antibiotic susceptibility and transferable elements requires further validation. Overall, the strain *W. cibaria* UF-274 is a promising candidate for functional food applications such as a starter/adjunct to enrich GABA in dairy products.

## Figures and Tables

**Table 1:** Analysis of variance (ANOVA) for GABA production.

Source	Sum of Squares	df	Mean Square	F-Value	*p*-Value	Significance
Model	52.05	9	5.78	8.23	0.0092	Significant
A-Glucose	0.2781	1	0.2781	0.3959	0.5524	
B-MSG	30.24	1	30.24	43.03	0.0006	
C-PLP	0.1931	1	0.1931	0.2748	0.6189	
AB	0.8798	1	0.8798	1.25	0.3059	
AC	2.06	1	2.06	2.93	0.1378	
BC	1.54	1	1.54	2.19	0.1896	
A2	10.70	1	10.70	15.23	0.0080	
B2	4.14	1	4.14	5.89	0.0514	
C2	2.03	1	2.03	2.89	0.1402	
Residual	4.22	6	0.7026			
Lack of fit	2.84	3	0.9479	2.07	0.2823	Not significant
Pure error	1.37	3	0.4572			
Cor total	56.26	15				

R^2^ = 92.51%.

**Table 2:** Genomic features of *W. cibaria* UF-274.

Attributes	Value
Contigs	4
Genome length	2,527,341
GC content	44.85584
Contig L50	1
Contig N50	2,460,442
tRNA	87
rRNA	28
CDS	2378
CDS ratio	0.94090
Hypothetical CDS	864
Predicted roles	1577

## Data Availability

The data presented in this study are available on request from the corresponding author.

## Abbreviations

The following abbreviations are used in this manuscript:

ANOVA	Analysis of Variance
BCP	Bromocresol Purple
BGC	Biosynthetic Gene Cluster
BUSCO	Benchmarking Universal Single-Copy Orthologs
CAZymes	Carbohydrate-Active enZymes
CDS	Coding Sequence
CRISPR	Clustered Regularly Interspaced Short Palindromic Repeats
GC	Guanine–Cytosine (content)
GABA	γ-Aminobutyric Acid
GAD	L-glutamic acid decarboxylase
gDNA	Genomic DNA
HPLC	High-Performance Liquid Chromatography
IS	Insertion Sequence
KEGG	Kyoto Encyclopedia of Genes and Genomes
MIC	Minimum Inhibitory Concentration
MSG	Monosodium Glutamate
N50	Contig length at which 50% of the genome assembly is contained
ONT	Oxford Nanopore Technologies
OPA	o-Phthaldialdehyde
ORF	Open Reading Frame
PLP	Pyridoxal-5′-phosphate
PKS	Polyketide synthase
RSM	Response Surface Methodology
RNA	Ribonucleic Acid
rRNA	Ribosomal RNA
T3PKS	Type III Polyketide Synthase
TLC	Thin-Layer Chromatography
WGS	Whole Genome Sequencing
